# Limit cycles and chaos in the hybrid atom-optomechanics system

**DOI:** 10.1038/s41598-022-15249-9

**Published:** 2022-09-10

**Authors:** Xingran Xu, Tanjung Krisnanda, Timothy C. H. Liew

**Affiliations:** 1 Division of Physics and Applied Physics, School of Physical and Mathematical Sciences, Nanyang Technological University, Singapore, 637371 Singapore; 2 MajuLab, International Joint Research Unit UMI 3654, CNRS, Université Côte d’Azur, Sorbonne Université, National University of Singapore, Nanyang Technological University, Singapore, Singapore

**Keywords:** Quantum optics, Ultracold gases, Theoretical physics

## Abstract

We consider atoms in two different periodic potentials induced by different lasers, one of which is coupled to a mechanical membrane via radiation pressure force. The atoms are intrinsically two-level systems that can absorb or emit photons, but the dynamics of their position and momentum are treated classically. On the other hand, the membrane, the cavity field, and the intrinsic two-level atoms are treated quantum mechanically. We show that the mean excitation of the three systems can be stable, periodically oscillating, or in a chaotic state depending on the strength of the coupling between them. We define regular, limit cycle, and chaotic phases, and present a phase diagram where the three phases can be achieved by manipulating the field-membrane and field-atom coupling strengths. We also computed other observable quantities that can reflect the system’s phase such as position, momentum, and correlation functions. Our proposal offers a new way to generate and tune the limit cycle and chaotic phases in a well-established atom-optomechanics system.

## Introduction

The hybrid atom-optomechanics system has been exploited due to its rich physics that allows for many opportunities, from theoretical proposals to experimental implementations. The frequent configuration of the system consists of a mechanical membrane (oscillator) and a Bose-Einstein condensate (BEC) that are mutually coupled to cavity field modes^1–4^. Applications resulting from this system have been valuable. For example, the mechanical oscillator can be cooled down by enhancing the effective coupling strength between the membrane and the atom^1,2,5–8^. At the same time, the BECs can have a nonequilibrium phase transition from the normal phase to the self-organized super-radiant phase^9–14^ due to the $Z_2$ symmetry breaking^15^. The system is also applicable for metrology^16–18^ and quantum simulations^19,20^. Last but not least, it provides a new platform to create new states of many-body physics, such as the spontaneous crystallization of atoms and light into a structure that features phonon-like excitations and bears similarities to a supersolid^21–25^. This motivates the further study of this system to potentially realize marvelous dynamical phases such as time crystal and chaos.

The time crystal phase breaks the time-translation symmetry^26,27^, which is beyond the strict thermal equilibrium^28–31^. Indeed, while quantum time crystals were originally defined as systems whose lowest energy state undergoes periodic motion, the definition has been extended to include nonlinear driven-dissipative systems^32,33^. Time crystals have been observed in many nonequilibrium experiments such as driven disordered dipolar spin impurities in diamonds^34^, the interacting spin chain of trapped atomic ions^35^, quantum computing processors^36^, etc. On the other hand, the chaotic phase represents unpredictable results after a long evolution time, which are sensitive to initial states^37^. In this case, chaotic attractors may arise, leading to orbits that converge to the corresponding chaotic region in the phase-space diagram^38,39^. For optomechanical system, chaotic dynamics appears in the bad-cavity limit and is described by the semiclassical equations of motion^40–42^. Limit cycles have been studied extensively in the atom-membrane setup, and observed experimentally^8,43^. Also for similar setups, limit cycles and the onset of chaos have been studied, for instance including work^44–46^. The hybrid atom-optomechanics system can be conditioned such that it satisfies the requirements for both limit cycle and chaotic phases, which urges a proposal for their realization and transition.

Experimental implementations have been reported for the atom-optomechanics system with $^{87}$Rb atoms and Si$_3$N$_4$ or SiN membrane^1,47^, where the position of the membrane displaces the lattice potential for the atoms^48–51^. Meanwhile, the center-of-mass motion of the atoms will experience a restoring optical dipole force due to the absorption and stimulated emission^9,10,47,52^. The optical lattice for the atoms can be highly engineered with different potentials. The depth of the potential can be adjusted by the power of the laser, while the period can be tuned by changing the wavelength of the laser or the angle between two beams^19,48,53^. The effective coupling between the atoms and the membrane can be long-distance interaction mediated by the laser field. The field interacts with the atoms via light-matter coupling, with an effective strength enhanced by the number of atoms ($\sim 10^{10}$)^1,7^.

In this paper, we consider the atoms trapped in a double well-like potential created by two lasers, where the wavelength of one is half of the other. One of the lasers is filtered and enters a cavity where it couples to a membrane, which in turn affects its optical path. This way, the atoms will have both time-dependent and fixed potentials. In this configuration, relevant interactions include optomechanical coupling between the field and the membrane as well as light-matter coupling between the field and the atoms. The position and momentum of the atoms are treated classically, with their dynamical equations coupled to a quantum master equation characterizing the membrane, the cavity field, and the intrinsic degrees of freedom of the atoms (two-level systems). We show that by tuning the strength of the optomechanical and light-matter coupling, the system can be in regular, limit cycle, or chaotic phases. We also computed experimentally familiar quantities such as the first and second-order correlation functions in different phases.

## Model

**Figure 1:**
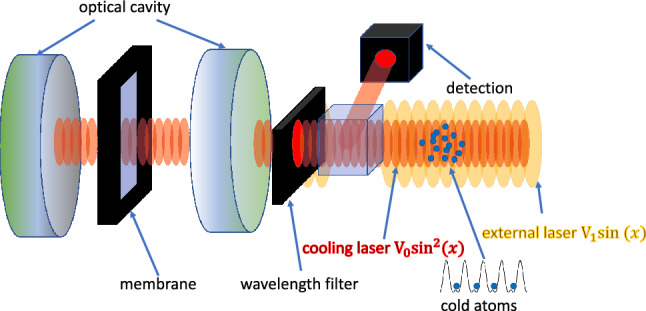
The scheme of the hybrid atom-optomechanics system. The cold atoms are trapped by two lasers and only one of them is filtered to enter a cavity where it is coupled to a mechanical membrane.

Consider two-level atoms moving in an adjusted gauge field optical lattice, which is coupled to a membrane through the coherently driven cavity field as shown in Fig. 1. The cold atoms are trapped by two lasers with different wavelengths, giving two optical lattice potentials with different periods. The Hamiltonian ($\hbar =1$) describing the membrane, cavity field, and two-level atom, in a frame rotating with the driving frequency $\omega _l$ and with rotating-wave approximation, is written as

1 $$\begin{aligned} H= & {} \omega _m {\hat{b}}^{\dagger }{\hat{b}}-\Delta _c {\hat{a}}^\dagger {\hat{a}} - \Delta _a{\hat{\sigma }}^+ {\hat{\sigma }}^- +\eta ({\hat{a}} + {\hat{a}}^\dagger )-g_{mc}({\hat{b}}^\dagger +{\hat{b}}){\hat{a}}^{\dagger }{\hat{a}} + g_{ac}\sin (2x)({\hat{a}}^\dagger {\hat{\sigma }}^- + {\hat{a}}{\hat{\sigma }}^+), \end{aligned}$$

where $\omega _m$ is the frequency of the membrane, $\Delta _c = \omega _l - \omega _c$ and $\Delta _a=\omega _l - \omega _a$ are the detuning for the cavity and atom, respectively. $\omega _c$ denotes the cavity frequency, $\omega _a$ the atomic transition frequency and $\omega _l$ the frequency of the laser driving the cavity with strength $\eta$. The atom couples to the cavity (Jaynes-Cummings type) with strength $g_{ac}$, while the optomechanical coupling^18^ between the membrane and cavity is denoted by $g_{mc}$. The optical lattice has a mode function $\sin (2x)$, where *x* is the atomic position, which is in units of the inverse cavity wave number. The annihilation operators for the cavity, atom, and membrane are denoted by ${\hat{a}}$, ${\hat{\sigma }}^-$, and ${\hat{b}}$, respectively.

The decays in the system are modelled by the Liouvillians and can be considered as Lindblad terms

2 $$\begin{aligned} \mathscr {L}_{\mu }[\rho ,{\hat{O}}] = \gamma _{\mu }(2{\hat{O}}\rho {\hat{O}}^\dagger - {\hat{O}}^\dagger {\hat{O}} \rho - \rho {\hat{O}}^\dagger {\hat{O}}), \end{aligned}$$

where $\gamma _{\mu }$ is the dissipation rate of the membrane ($\mu =m$), cavity field (*c*), and two-level atom (*a*). Note that ${\hat{O}}$ denotes the corresponding annihilation operator of each system. As the initial state, we use uncorrelated states of the form $\rho =\rho _m\otimes \rho _c\otimes \rho _a$, where $\rho _m$, $\rho _c$, and $\rho _a$ represent the density matrix for the membrane, cavity field, and atom. The evolution follows the quantum master equation:

3 $$\begin{aligned} {{\dot{\rho }}}=-i[H,\rho ]+\frac{1}{2}\left( \mathscr {L}_{m}[\rho ,{\hat{b}}]+\mathscr {L}_{c}[\rho ,{\hat{a}}]+\mathscr {L}_{a}[\rho ,{\hat{\sigma }}^-]\right) . \end{aligned}$$

In addition, we have classical differential equations of the atomic motion obtained from the Ehrenfest theorem: $\dot{x}={\partial \langle H\rangle }/{\partial p}$ and $\dot{p}=-{\partial \langle H\rangle }/{\partial x}$, where the observables *x* and *p* are treated simply as numbers in the classical regime. For this classical motion, the atom is situated in two potentials such that its Hamiltonian reads

4 $$\begin{aligned} H_a=\frac{p^2}{2m}+V_0\sin (x)^2+V_1 \sin (x), \end{aligned}$$

where *m* is the mass of the atom, $V_0$ is the depth of the optical lattice, and $V_1 \sin (x)$ is the external periodic potential. We stress that only one of the potentials ($V_0$) ends up being coupled to the membrane. The equations of motion for the atom, taking into account the Hamiltonians *H* and $H_a$, are written as

5 $$\begin{aligned} \dot{x}= & {} 2\omega _r p, \nonumber \\ \dot{p}= & {} -4g_{ac}\cos (2x)\,\Re \left\{ \langle {\hat{a}}^\dagger {\hat{\sigma }}^-\rangle \right\} -\left[ V_1 \cos (x)+V_0\sin (2x)\right] , \end{aligned}$$

where $\omega _r=1/(2m)$ is the recoil frequency. As initial conditions, we take $x(0)=-1$ and $p(0)=0$. We note that the quantum observable $\Re \left\{ \langle {\hat{a}}^\dagger {\hat{\sigma }}^-\rangle \right\}$ updates the classical dynamics, while the latter affects the quantum dynamics via the change in the optical path, and hence, the mode function $\sin (2x)$. We show below with suitable parameters, that this quantum-classical coupled dynamics can produce regular, limit cycle, and chaotic phases. See also the Appendix for calculations of the dynamics using the quantum trajectory method.

## Different dynamical behaviors

**Figure 2:**
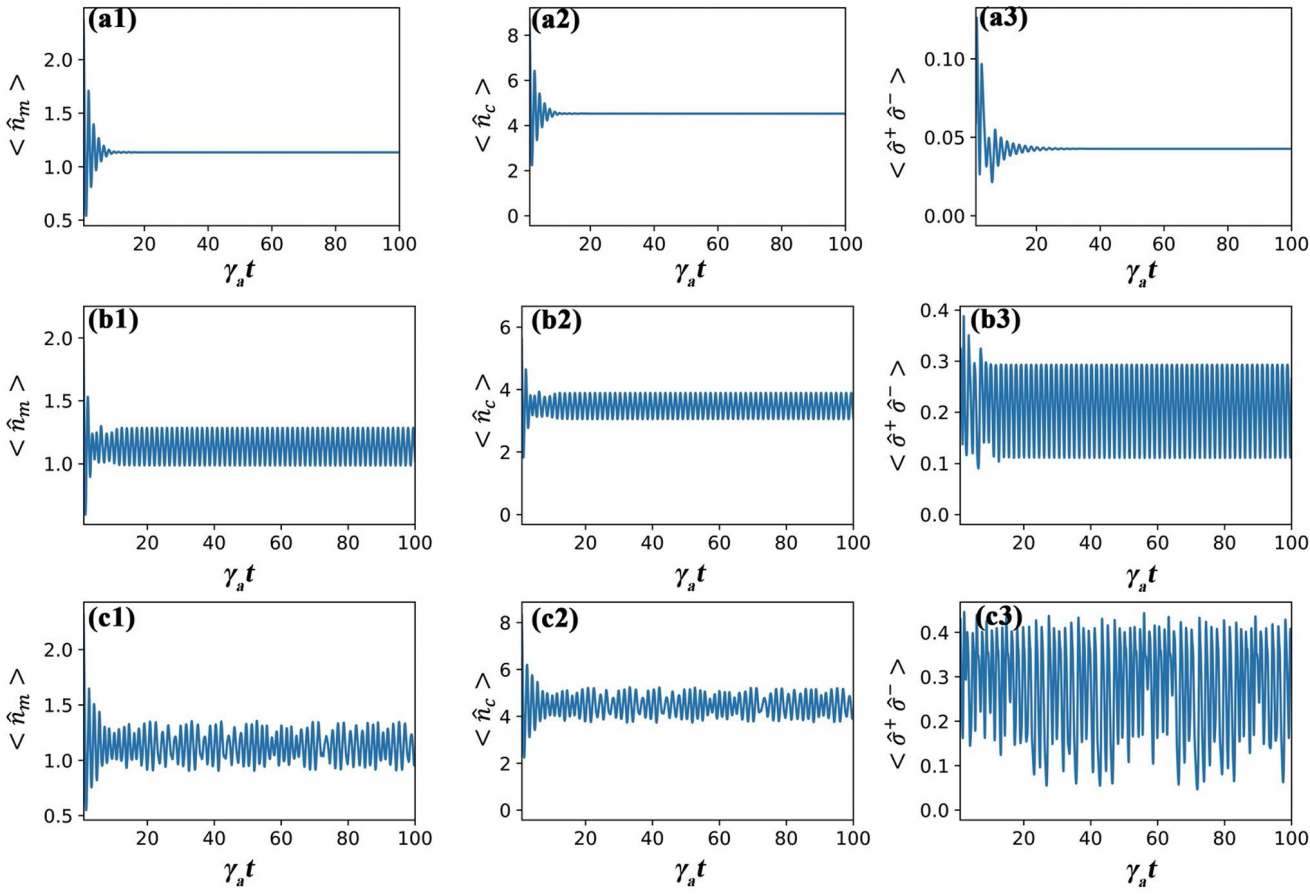
The mean excitation of the membrane mode (first column), the cavity mode (second column) and the atom (third column). The parameters used are $\gamma _c/\gamma _a$ = 0.5, $\gamma _m/\gamma _a$ = 2, $\eta /\gamma _a$ = 5, $V_0/\gamma _a$ = 20, $V_1/\gamma _a$ = 40, $\omega _r/\gamma _a$ = 1, $\Delta _c/\gamma _a$ = − 1, and $\Delta _a/\gamma _a$ = − 2. Specific coupling parameters for different phases are given by (**a1**)–(**a3**) $g_{ac}/\gamma _a$ = 0.5, $g_{mc}/\gamma _a$ = 2; (**b1**)–(**b3**) $g_{ac}/\gamma _a$ = 2, $g_{mc}/\gamma _a$ = 2.5; and (**c1**)–(**c3**) $g_{ac}/\gamma _a$ = 2, $g_{mc}/\gamma _a$ = 2.

**Figure 3:**
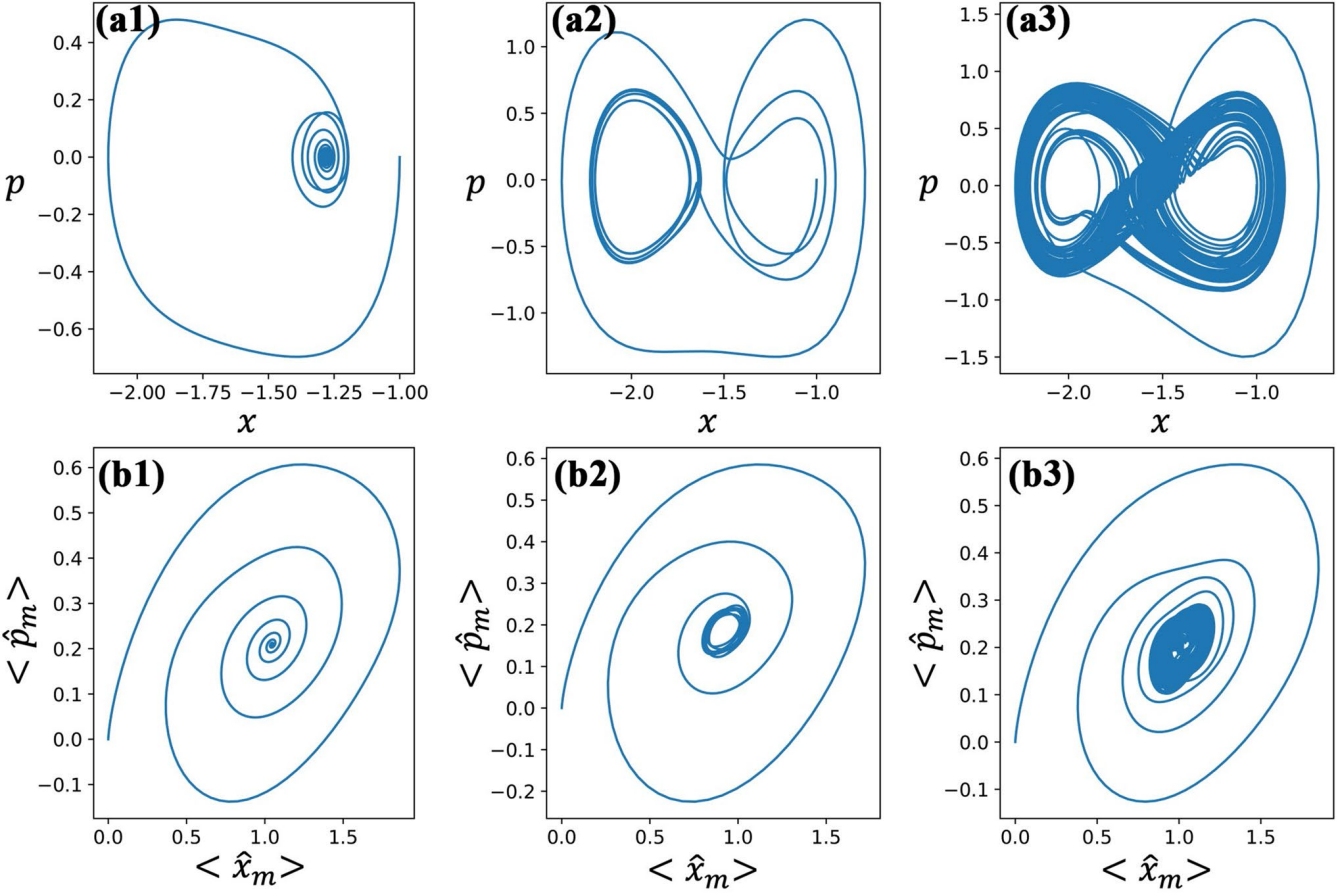
The motion of the atom (first row) and the membrane (second row) in the phase-space diagram. The parameters are given by $\gamma _c/\gamma _a$ = 0.5, $\gamma _m/\gamma _a$ = 2, $\eta /\gamma _a$ = 5, $V_0/\gamma _a$ = 20, $V_1/\gamma _a$ = 40, $\omega _r/\gamma _a$ = 1, $\Delta _c/\gamma _a$ = − 1, and $\Delta _a/\gamma _a$ = − 2. For panels (**a1**,**b1**): $g_{ac}/\gamma _a$ = 0.5, $g_{mc}/\gamma _a$ = 2; (**a2**,**b2**): $g_{ac}/\gamma _a$ = 2, $g_{mc}/\gamma _a$ = 2.5; and (**a3**,**b3**): $g_{ac}/\gamma _a$ = 2, $g_{mc}/\gamma _a$ = 2.

The hybrid system has quantum and classical parts that are treated differently but are coupled to each other. For the quantum part, all three systems are also coupled, and consequently, we note that a particular phase in one system is an indication of the same phase in others. In what follows, we define three phases based on the dynamical behavior of mean excitations (either of the membrane $\langle n_{m}\rangle$, cavity field $\langle n_{c}\rangle$, or two-level atom $\langle {\hat{\sigma }}^+ {\hat{\sigma }}^- \rangle$):

1. *Regular phase.* The excitation of each system will have a stable value after a long evolution time, as shown in Fig. 2a1–a3. In this case, the motion of the atom and the membrane will converge to a point in the phase-space diagram, as shown in Fig. 3a1,b1.
2. *Limit cycle phase.* The excitation of each system will oscillate periodically around a certain value, as shown in Fig. 2b1–b3. Here, after a certain time, the motion of the atom and the membrane will continue to orbit a point in the phase-space diagram, see Fig. 3a2,b2.
3. *Chaotic phase.* The excitation of each system will show random oscillation, as shown in Fig. 2c1–c3. The motion of the atom and the membrane in the phase-space diagram will exhibit random orbits around two attractors, see Fig. 3a3,b3.

Remarkably, the quantum-classical coupled dynamics can produce all three phases, by simply tuning the strength of the optomechanical ($g_{mc}$) and light-matter ($g_{ac}$) coupling, see Fig. 2.

Furthermore, the atomic motion (*x*, *p*) and expectation value of quadratures, e.g., for the membrane $(x_m, p_m)$, where $x_{m}=\langle {\hat{b}}+{\hat{b}}^{\dagger }\rangle /\sqrt{2}$ and $p_{m}=\langle {\hat{b}}-{\hat{b}}^{\dagger }\rangle /(i\sqrt{2})$ are plotted in phase-space diagrams in Fig. 3. For the atom, as the period of one potential is twice the other (see Eq. (4)), the depths $V_0$ and $V_1$ allow for a double well-like potential shape, which consequently gives three optimum points, two of which are stable. The position of the two stable points are symmetric with respect to, e.g., $x=-\pi /2$ where the stronger potential $V_1\sin (x)$ has the lowest energy. Thus, the steady momentum is always zero in the regular phase, whereas it is oscillating around zero in other phases. The atomic motion will converge to one of the stable points in the regular phase while the trajectory will form a closed circle in the limit cycle phase. When the system is in the chaotic phase, there are two attractors in the phase-space diagram and the motion of the atom is unpredictable. At the same time, the behaviors of the quantum degrees of freedom reflect that of the classical ones (*x*, *p*) of the atom, see the second column of Fig. 3 for the membrane’s quadratures. The motion of the membrane will have non-zero momentum in the steady-state regime. Below we shall introduce quantities to indicate the phase of the system, and finally, obtain a phase transition diagram for varying values of the optomechanical and light-matter coupling strengths.

We also computed the first and second order correlation functions $G^{(1)}(\tau )$ and $G^{(2)}(\tau )$ that are standardly measured in experiments. See the Appendix for details. As expected, the behaviors of these correlation functions follow that of the mean excitation in the corresponding phases.

## Quantification and classification of the phases

Here we shall present a way to numerically classify the phases previously described. In particular, we used two quantities, where one is recognizing the regular phase and the other the chaotic phase. Consequently, this method classifies all three possible phases in the phase transition diagram, which we will present below.

### The regular phase transition

Recognizing the regular phase is straightforward as the mean excitation of all the systems will go towards a constant value, see Fig. 2. Here, after a long evolution time, one can choose a time range and compute $R=\max {(\langle n_{\mu }\rangle )}-\min {(\langle n_{\mu }\rangle )}$. The regular phase is given for $R<\epsilon$, where $\epsilon$ is a small constant.

### The 0-1 test for the chaotic phase transition

**Figure 4:**
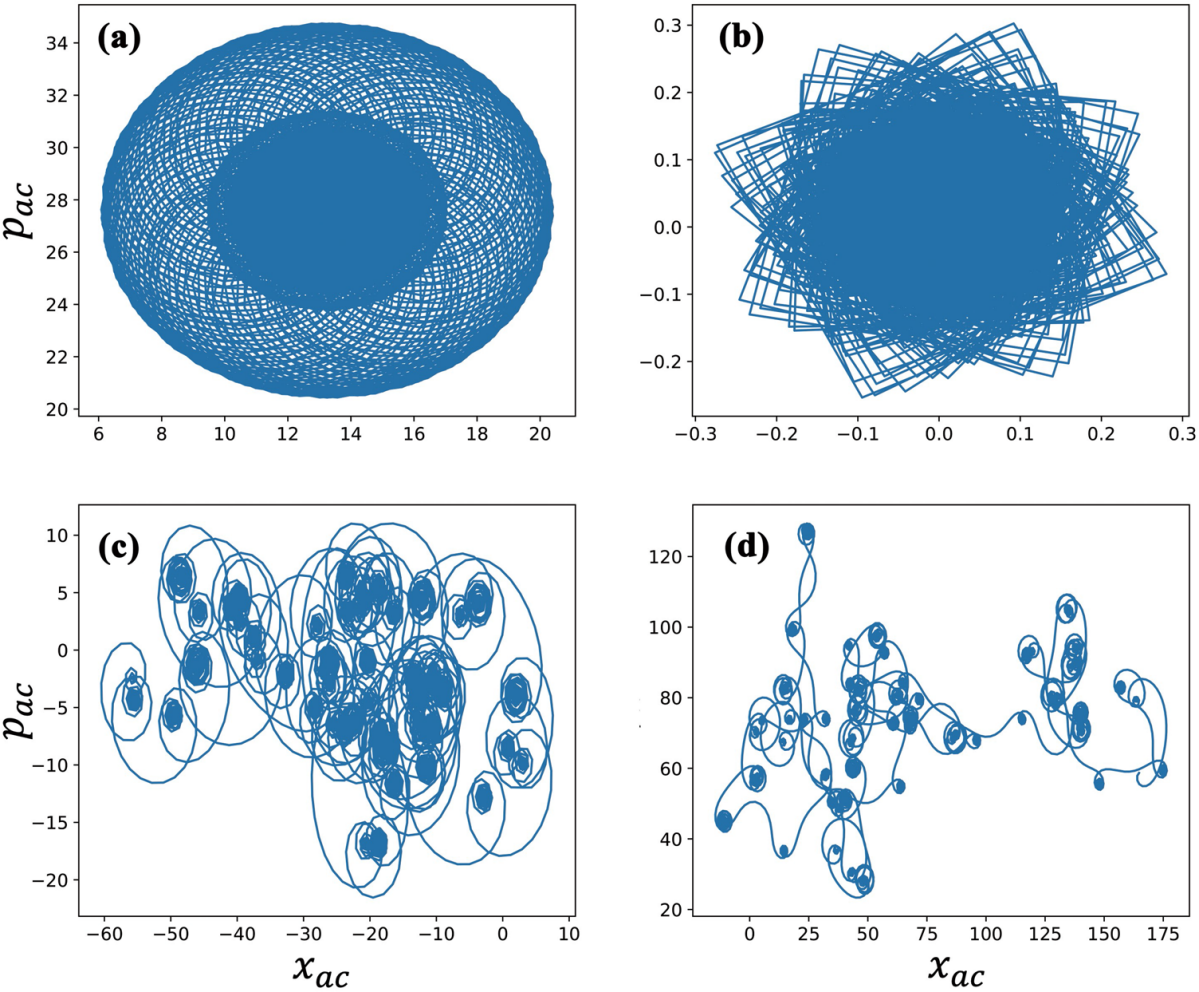
The dynamics of the translation components $(x_{ac},\,p_{ac})$. The parameters used are $\gamma _c/\gamma _a$ = 0.5, $\gamma _m/\gamma _a$ = 2, $\eta /\gamma _a$ = 5, $V_0/\gamma _a$ = 20, $V_1/\gamma _a$ = 40, $\omega _r/\gamma _a$ = 1, $\Delta _c/\gamma _a$ = − 1, and $\Delta _a/\gamma _a$ = − 2. We also used (**a**) $g_{ac}/\gamma _a$ = 0.5, $g_{mc}/\gamma _a$ = 2; (**b**) $g_{ac}/\gamma _a$ = 2, $g_{mc}/\gamma _a$ = 2.5; (**c**) $g_{ac}/\gamma _a$ = 2, $g_{mc}/\gamma _a$ = 2; and (**d**) $g_{ac}/\gamma _a$ = 4, $g_{mc}/\gamma _a$ = 2.

The system in the chaotic phase will have a very different dynamical behavior, which can be tested by the regression or correlation method^54^. Here, relevant functions are defined such that we can apply the above tests to our system. First, we take new translation components ($$x_{ac}$$, $$p_{ac})$$ and $$\theta _c$$ as follows

6 $$\begin{aligned} p_{ac}(n+1)= & {} \phi (n) \cos (\theta _c)+p_{ac}(n),\nonumber \\ x_{ac}(n+1)= & {} \phi (n)\sin (\theta _c)+x_{ac}(n+1),\nonumber \\ \theta _c(n+1)= & {} \nu +\theta _c(n)+\phi (n), \end{aligned}$$

where $n=1,2,\ldots , N$ denotes the time index, $\phi (n)$ is a dynamical quantity, here taken as $x(n)+p(n)$, and $\nu$ is a fixed constant $[0,\pi ]$. The initial state of $p_{ac}$, $x_{ac}$ and $\theta _c$ are zero and they are updated by the position and the momentum of atoms. The quantities $q_{ac}$ and $p_{ac}$ are bounded if the dynamical behavior is regular, while in the chaotic phase they will behave asymptotically. The translation components resulting from the hybrid atom-optomechanical system are shown in Fig. 4. The regular and limit cycle phases have bounded states for ($x_{ac},p_{ac}$) as shown in Fig. 4a,b. However, they become unbounded in the chaotic phase, see Fig. 4c,d, showing the pattern of fractals.

Given dynamical components ($x_{ac},p_{ac}$), the mean square displacement is defined as

7 $$\begin{aligned} M_c(n)= & {} \lim _{N\rightarrow \infty }\frac{1}{N}\sum _{j=1}^{N}\left[ p_{ac}(j+n)-p_{ac}(j) \right] ^2+\left[ x_{ac}(j+n)-x_{ac}(j) \right] ^2, \end{aligned}$$

where $n\ll N$ is required. The test for chaos is based on the growth rate of $M_c(n)$ as a function of *n*. A modified mean square displacement $D_c(n)$ that exhibits the same asymptotic growth as $M_c(n)$, but with better convergence properties is given by

8 $$\begin{aligned} D_c(n)=M_c(n)-V_{osc}(\nu ,n), \end{aligned}$$

where the oscillation term $V_{osc}$ is defined as $V_{osc}=(E_\phi )^2({1-\cos (n\nu )})/({1-\cos (\nu )}),$ and the expectation $E_\phi$ is given by $E_\phi =\lim _{N\rightarrow \infty }\frac{1}{N}\sum _{j=1}^N \phi (j)$. Note that the cut-off index $n_{\text {cut}}$ needs to be large enough such that the error of $D_c(n_{\text {cut}})$ is close to zero.

The 0-1 test via regression method is calculated following the quantity $K_c=\lim _{n\rightarrow \infty }{\log M_c(n) }/{\log n}$, whose value is near zero (one) for non-chaotic (chaotic) phase. An alternative test, that we also consider, is via the correlation method^54^ and it is determined by the mean square displacement $D_c$ as follows

9 $$\begin{aligned} \text {cov}(X,Y)\equiv & {} \frac{1}{q}\sum _{j=1}^{q}\left( X(j)-\bar{X} \, \right) \left( Y(j)-\bar{Y} \right) , \nonumber \\ K_c= & {} \frac{\text {cov}(\xi ,\Delta )}{\sqrt{\text {cov}(\xi ,\xi )\text {cov}(\Delta ,\Delta )}}, \end{aligned}$$

where $\bar{X}$ and $\bar{Y}$ are the mean values of the vectors *X* and *Y* with length *q*. We take the vectors $\xi =(1,2,\ldots ,n_{\text {cut}})$ and $\Delta =(D_c(1), D_c(2),\ldots ,D_c(n_{\text {cut}}))$.

### The phase transition diagram

**Figure 5:**
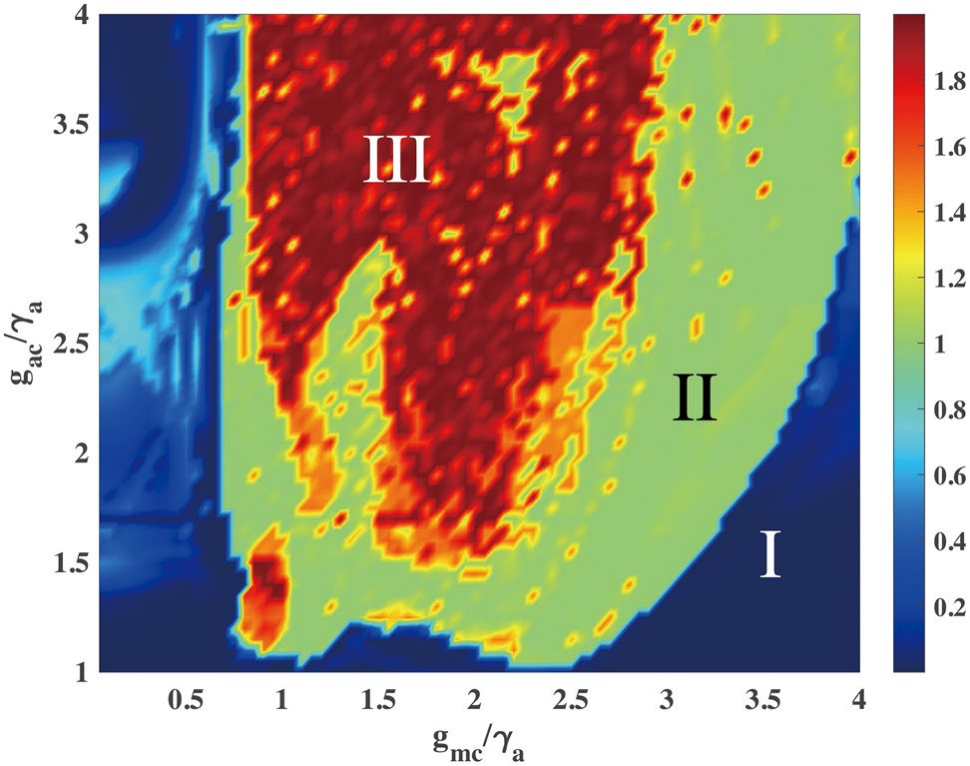
The phase transition diagram. The regular, limit cycle and chaotic phases are denoted by I, II and III, respectively. The parameters used are $\gamma _c/\gamma _a$ = 0.5, $\gamma _m/\gamma _a$ = 2, $\eta /\gamma _a$ = 5, $V_0/\gamma _a$ = 20, $V_1/\gamma _a$ = 40, $\omega _r/\gamma _a$ = 1, $\Delta _c/\gamma _a$ = − 1, and $\Delta _a/\gamma _a$ = − 2.

The three phases are characterized by the two tests described above (regular and chaotic phase transition tests). The phase diagram for the two tests are plotted separately, see the Appendix. Here, we combine the diagrams, see Fig. 5, which shows the three phases for different coupling strengths. When the atom-cavity coupling is close to zero, only the regular phase exists with the balance of the rates of the decay and the drive. With the increase of $g_{ac}$, the limit cycle phase will appear with periodic evolution of the interaction strength $g_{ac}\Re \left\{ \langle {\hat{a}}^\dagger {\hat{\sigma }}^-\rangle \right\}$. For further increase of $g_{ac}$ the system reaches the chaotic phase. Remarkably, the coupling between the cavity and the membrane $g_{mc}$ also plays an important role in the limit cycle and chaotic phases. If $g_{mc}$ is too small compared to $g_{ac}$ the model can be simplified to an atom cooling model and the membrane’s oscillations can be ignored. On the contrary, if $g_{ac}$ is too small, the system can be transformed to an optomechanical model and the atoms can be ignored. The competition of the coupling strengths allows the system to have a rich phase diagram. The regular-limit cycle transitions start to happen at $g_{mc}/\gamma _a \sim 0.8$ and $g_{ac}/\gamma _a \sim 1$, see Fig. 5. In other words, exemplary parameters for obtaining the regular phase are $g_{mc}/\gamma _a<0.8$ and $g_{ac}/\gamma _a<1$. For observing the other two phases, exemplary parameters are given as follows. For the limit cycle: $g_{mc}/\gamma _a=3$ and $g_{ac}/\gamma _a=2$, while for the chaotic phase: $g_{mc}/\gamma _a=1$ and $g_{ac}/\gamma _a=3$.

## Conclusion

We theoretically considered a hybrid atom-optomechanics system to realize different dynamical phases by exploiting the competition of the coupling strength of the cavity and atoms, and that of the cavity and membrane. The atoms experience two potentials, including one that may be static, periodically oscillating, or randomly oscillating. The coupling of the cavity mode and the membrane allows them to have similar behavior, where the whole system can exhibit a regular, limit cycle, or chaotic phase. These three phases are distinguished after evolving quantities from the system for a sufficiently long time, where we performed regular and chaotic phase transition tests. Our study motivates possibilities such as indirect measurements or inference of the states of the membrane from the states of the atoms, or vice versa. It is also interesting to study the dynamics of quantum correlations (e.g., entanglement) between different subsystems in different phases for potential quantum information applications such as quantum state transfer and metrology similar to what has been achieved in optomechanics^18^.

## Supplementary Information

Supplementary Information.
