# Supplementary material for: Limit cycles and chaos in the hybrid atom-optomechanics system
Source: Sci Rep. 2022 Sep 10;12:15288. doi: 10.1038/s41598-022-15249-9 (PMC9464193; doi:10.1038/s41598-022-15249-9)
Supplement: Supplementary file 1 — Supplementary Information. [file 41598_2022_15249_MOESM1_ESM.pdf]

Supplementary Information: Limit cycles and chaos in the hybrid atom-optomechanics system

Xingran Xu[1], Tanjung Krisnanda[1], and Timothy C. H. Liew[1,2]

[1]Division of Physics and Applied Physics, School of Physical and Mathematical Sciences, Nanyang Technological University, Singapore 637371, Singapore
[2]MajuLab, International Joint Research Unit UMI 3654, CNRS, Université Côte d'Azur, Sorbonne Université, National University of Singapore, Nanyang Technological University, Singapore

S1. Quantum trajectory method

We also use the quantum trajectory (QT) method as a separate way to evolve the atom-optomechanics system. For a review on quantum trajectories, see Refs.[1,2]. As described in the main text, the evolution of the system is governed by the coupled quantum-classical dynamics. The quantum dynamics is described within the quantum master equation, which here we describe using the QT method. The observable $\Re\left\{\langle\hat{a}^\dagger\hat{\sigma}^-\rangle\right\}$ obtained from the QT will then update the classical dynamics for the atomic motion, which in turn affects the Hamiltonian (via $\sin(2x)$) of all the trajectories.

We begin by noting that the quantum master equation in Eq. (3) can be rewritten as

$$\dot{\rho} = -i(H_{\mathrm{eff}}\rho - \rho H_{\mathrm{eff}}^\dagger) + \tilde{b}\rho\tilde{b}^\dagger + \tilde{a}\rho\tilde{a}^\dagger + \tilde{\sigma}^-\rho\tilde{\sigma}^+, \tag{S1}$$

where $H_{\mathrm{eff}} = H - (i/2)(\tilde{b}^\dagger\tilde{b} + \tilde{a}^\dagger\tilde{a} + \tilde{\sigma}^+\tilde{\sigma}^-)$ and the decay rates are absorbed into the operators, i.e., $\tilde{b} = \sqrt{\gamma_m}\hat{b}$, $\tilde{a} = \sqrt{\gamma_c}\hat{b}$, and $\tilde{\sigma}^- = \sqrt{\gamma_a}\hat{\sigma}^-$. The interpretation of Eq. (S1) is that the system is evolved under H_{eff} and at the same time possible *jumps* may occur, from the rest of the terms. This way, the evolution of each trajectory from t to $t+\delta t$ is constructed as follows. A candidate state is calculated as $|\psi^{(1)}(t+\delta t)\rangle = (1 - iH_{\mathrm{eff}}\delta t)|\psi(t)\rangle$. As H_{eff} is not Hermitian, one obtains

$$\langle\psi^{(1)}(t+\delta t)|\psi^{(1)}(t+\delta t)\rangle = 1 - \delta p, \tag{S2}$$

where δp is a probability. One can further note that

$$\begin{aligned}\delta p &= \delta t\langle\psi(t)|i(H_{\mathrm{eff}} - H_{\mathrm{eff}}^\dagger)|\psi(t)\rangle \\ &= \delta t\langle\psi(t)|\tilde{b}^\dagger\tilde{b} + \tilde{a}^\dagger\tilde{a} + \tilde{\sigma}^+\tilde{\sigma}^-|\psi(t)\rangle \\ &= \delta p_m + \delta p_c + \delta p_a,\end{aligned} \tag{S3}$$

where we have used, e.g., $\delta p_m \equiv \delta t\langle\psi(t)|\tilde{b}^\dagger\tilde{b}|\psi(t)\rangle$. The stochastic evolution step is computed as follows:

1. With probability $1-\delta p$, the new state is
$$|\psi(t+\delta t)\rangle = \frac{|\psi(t+\delta t)\rangle}{\sqrt{1-\delta p}}. \tag{S4}$$

2. With probability δp, one of the jumps happens. The new state will be one of the following:

$$\begin{aligned}|\psi(t+\delta t)\rangle &= \frac{\tilde{b}|\psi(t)\rangle}{\sqrt{\delta p_m/\delta t}}; \\ |\psi(t+\delta t)\rangle &= \frac{\tilde{a}|\psi(t)\rangle}{\sqrt{\delta p_c/\delta t}}; \\ |\psi(t+\delta t)\rangle &= \frac{\tilde{\sigma}^-|\psi(t)\rangle}{\sqrt{\delta p_a/\delta t}}.\end{aligned} \tag{S5}$$

The probability of each state is proportional to δp_m, δp_c, and δp_a, respectively.

The expectation value of an observable is obtained from the average of all trajectories, e.g.,

$$\langle n_c(t)\rangle = \frac{1}{N}\sum_j^N\langle\psi_j(t)|\hat{a}^\dagger\hat{a}|\psi_j(t)\rangle, \tag{S6}$$

where $|\psi_j(t)\rangle$ is the state of the jth trajectory. For initial states that are mixed, pure states are sampled from the ones composing the initial density matrix, which are then evolved following the QT method.

We demonstrate the computation of $\langle n_c(t)\rangle$ with the QT method (using 1000 trajectories) in Fig. S1(a) and (b), where the initial states are taken as $|100\rangle$ and $|110\rangle$, respectively. It can be seen that the calculations from the QT method are close to that from the quantum master equation (solid black curves), as expected. The ratio of the mean excitation in panel (a) to panel (b) is simply the correlation function $G^{(2)}(\tau)$ (will be properly introduced later, see Eq. (S12)), where t is taken to be zero. For this example, $G^{(2)}(\tau)$ is plotted in panel Fig. S1 (c), where it oscillates around unity.

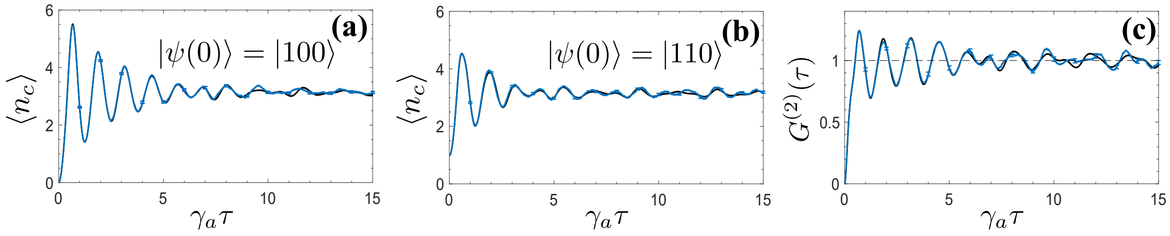

Figure S1. The evolution of mean excitation of the cavity field mode via the quantum trajectory method. Panels (a) and (b) represent evolution starting with different initial states. Panel (c) is the second order correlation function. The corresponding results using the quantum master equation are also plotted in each panel (solid black curves). The error bars represents the standard error of the mean from 1000 trajectories. The parameters used are γ_c/γ_a=0.5, γ_m/γ_a=2, η/γ_a=5, V_0/γ_a=20, V_1/γ_a=40, ω_r/γ_a=1, Δ_c/γ_a=-1, Δ_a/γ_a=-2, g_{ac}/γ_a=2, and g_{mc}/γ_a=2.5.

S2. The correlation functions

The correlation functions are normally used to describe coherence properties of electromagnetic fields. Here we shall compute these quantities for the cavity field mode of the atom-optomechanics system. The first and second order correlation functions are defined, respectively, as

$$G^{(1)}(\tau) = \frac{\langle \hat{a}^\dagger(t+\tau)\hat{a}(t)\rangle}{\sqrt{\langle n_c(t)\rangle\langle n_c(t+\tau)\rangle}}, \tag{S7}$$

$$G^{(2)}(\tau) = \frac{\langle \hat{a}^\dagger(t)\hat{a}^\dagger(t+\tau)\hat{a}(t+\tau)\hat{a}(t)\rangle}{\langle n_c(t)\rangle\langle n_c(t+\tau)\rangle}, \tag{S8}$$

where $\langle n_c(t)\rangle = \mathrm{Tr}\left[\hat{a}^\dagger\hat{a}\rho(t)\right]$ and $\langle n_c(t+\tau)\rangle = \mathrm{Tr}\left[\hat{a}^\dagger\hat{a}\rho(t+\tau)\right]$.

To calculate the numerator of the first order correlation function $G^{(1)}(\tau)$ in Eq. (S7), the initial density matrix $\rho(0)$ is evolved to $\rho(t)$ with the quantum-classical coupled dynamics. The subsequent evolution requires helper states, defined as

$$\begin{aligned}
\tilde{\rho}_1(t) &= (1+\hat{a})\rho(t)(1+\hat{a}^\dagger),\\
\tilde{\rho}_2(t) &= (1-\hat{a})\rho(t)(1-\hat{a}^\dagger),\\
\tilde{\rho}_3(t) &= (1+i\hat{a})\rho(t)(1-i\hat{a}^\dagger),\\
\tilde{\rho}_4(t) &= (1-i\hat{a})\rho(t)(1+i\hat{a}^\dagger).
\end{aligned} \tag{S9}$$

Note that this way, we have $(\tilde{\rho}_1(t)-\tilde{\rho}_2(t)-i\tilde{\rho}_3(t)+i\tilde{\rho}_4(t))/4 = \hat{a}\rho(t) \equiv \hat{A}(t)$. The normalised helper states ($\rho_j(t) = \tilde{\rho}_j(t)/\mathrm{Tr}\left[\tilde{\rho}_j(t)\right]$) are physical density matrices, which are then evolved from t to $t+\tau$. With this method, one obtains

$$\hat{A}(t+\tau) = \frac{1}{4}[\tilde{\rho}_1(t+\tau)-\tilde{\rho}_2(t+\tau)-i\tilde{\rho}_3(t+\tau)+i\tilde{\rho}_4(t+\tau)]. \tag{S10}$$

Finally, the first order correlation function is given by

$$G^{(1)}(\tau) = \mathrm{Tr}\left[\hat{a}^\dagger\hat{A}(t+\tau)\right]/\sqrt{\langle n_c(t)\rangle\langle n_c(t+\tau)\rangle}. \tag{S11}$$

The second order correlation function is computed in a similar way. After the first evolution, leading to $\rho(t)$, one considers a photon-subtracted state $\rho_p(t) = \hat{a}\rho(t)\hat{a}^\dagger/\langle n_c(t)\rangle$. This physical state is then evolved from t to τ, giving $\rho_p(t+\tau)$. The correlation function in Eq. (S8) is then evaluated as

$$G^{(2)}(\tau) = \mathrm{Tr}\left[\hat{a}^\dagger\hat{a}\rho_p(t+\tau)\right]/\langle n_c(t+\tau)\rangle. \tag{S12}$$

Recall that the denominator in Eq. (S12) is simply Tr $\left[\hat{a}^\dagger \hat{a} \rho(t+\tau)\right]$. Therefore, it is expected that in the regular phase, given large τ, the state $\rho_p(t+\tau) = \rho(t+\tau)$ is the steady state solution, making $G^{(2)}(\tau) = 1$. This is not the case for the limit cycle and chaotic phases, as the mean excitation still oscillates for large τ. In this case, the $G^{(2)}(\tau)$ will also oscillate and cross unity during its evolution.

As exemplary cases, we present the first (dashed blue curves) and second (solid orange curves) order correlation functions in Fig. S2. It can be seen that $G^{(2)}(\tau) \to 1$ in the regular phase, Fig. S2(a1), while it is oscillating around one in the limit cycle phase, as shown in Fig. S2(a2). This oscillation is also observed in the chaotic phase, but it is random, see Figs. S2(b1)-(b2).

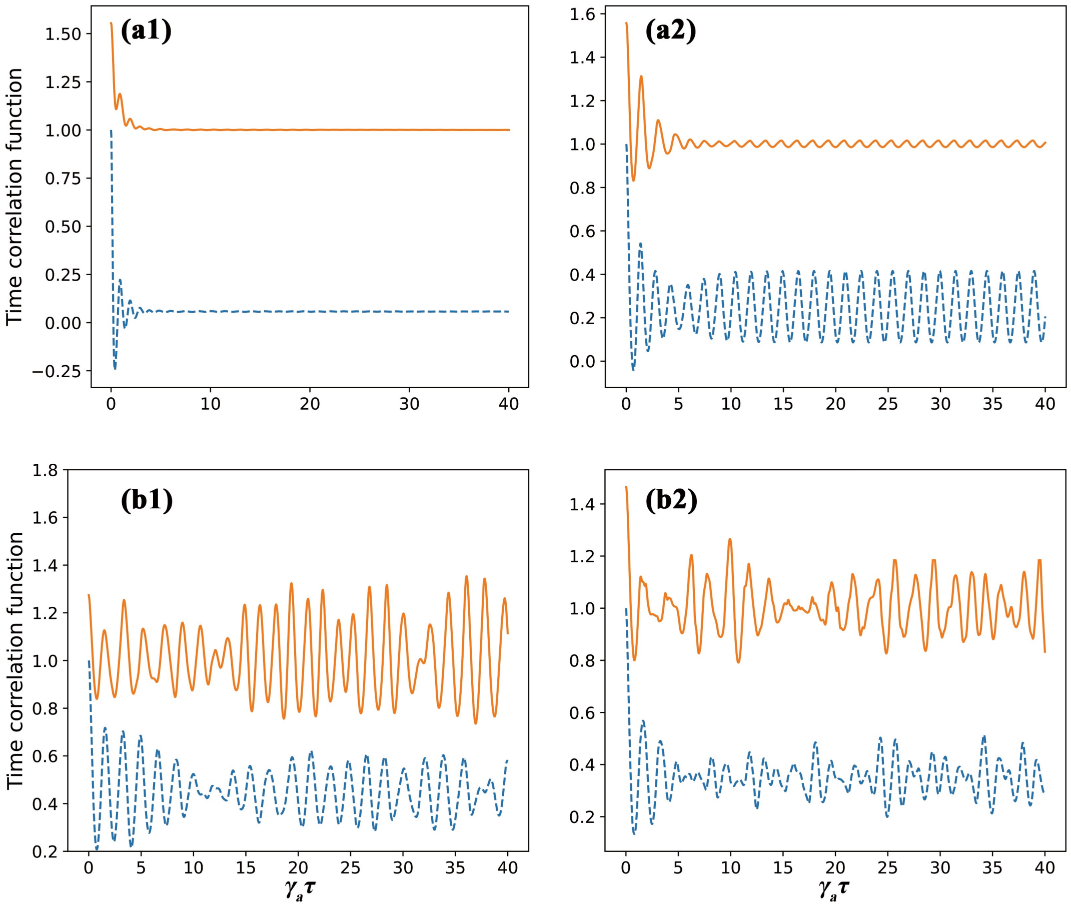

Figure S2. The first and second order correlation functions of the cavity mode, indicated by the dashed blue and solid orange curves, respectively. The parameters are summarised as follows γ_c/γ_a=0.5, γ_m/γ_a=2, η/γ_a=5, V_0/γ_a=20, V_1/γ_a=40, ω_r/γ_a=1, Δ_c/γ_a=-1, Δ_a/γ_a=-2, and for panel (a1): g_{ac}/γ_a=4, g_{mc}/γ_a=2; (a2): g_{ac}/γ_a=2, g_{mc}/γ_a=2.5; (b1): g_{ac}/γ_a=2, g_{mc}/γ_a=2; and (b2): g_{ac}/γ_a=4, g_{mc}/γ_a=2.

S3. The phase diagram for the regular and chaotic phase transition tests

The phase diagram in the main text (Fig. 5) is determined by two tests. The first one is the regular transition test where it recognizes the cavity mode's excitation converging to a certain value after a long evolution time. The second one is the chaos test, characterised by Eq. (9), where K_c will be close to 1 when the system is in a chaotic phase.

The regular phase transition is shown in Fig. S3 (a) with the blue region indicating the regular phase and the yellow one representing other phases. When the field-membrane coupling strength g_{mc} is small, there is only regular phase regardless of the field-atom coupling strength g_{ac}. Along with the increase of g_{mc}, the system can be in a chaotic or limit cycle phase. However, if g_{mc} is too large, the influence of the atom in the time dependent Hamiltonian can be ignored and the system returns to the regular phase.

The value of K_c is shown in Fig. S3 (b) and the chaotic phase, indicated by the redder region, can only exist in the yellow region of Fig. S3 (a). The chaotic phase appears in the region where $g_{ac}/\gamma_a > 1$ and $g_{mc}/\gamma_a \gtrsim 1$, i.e., the membrane and the

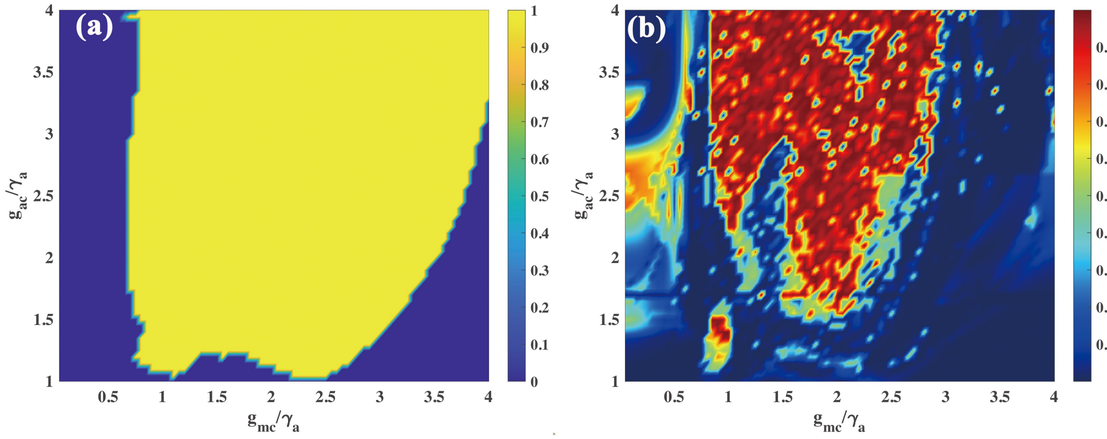

Figure S3. The regular phase transition test (a) and the correlation methods for the chaos test (b). The parameters used are γ_c/γ_a=0.5, γ_m/γ_a=2, η/γ_a=5, V_0/γ_a=20, V_1/γ_a=40, ω_r/γ_a=1, Δ_c/γ_a=-1, and Δ_a/γ_a=-2.

atoms both influence the the dynamical behavior of the whole system.

The sum of the values from Fig. S3 (a) and Fig. S3 (b) is plotted in Fig. 5 of the main text. The region I (blue color) indicates the regular phase and the region III the chaotic phase (red color). The limit cycle phase is then inferred from the remaining region, labelled II (green color).

References

1. Carmichael, H. *An open systems approach to quantum optics: lectures presented at the Université Libre de Bruxelles, October 28 to November 4, 1991*, vol. 18 (Springer Science & Business Media, 2009).
2. Daley, A. J. Quantum trajectories and open many-body quantum systems. *Adv. Phys.* **63**, 77–149 (2014).
